# Imaging Function and Relative Light Transmission of Explanted Opacified Hydrophilic Acrylic Intraocular Lenses

**DOI:** 10.3390/diagnostics13101804

**Published:** 2023-05-19

**Authors:** Tadas Naujokaitis, Ramin Khoramnia, Grzegorz Łabuz, Chul Young Choi, Gerd U. Auffarth, Tamer Tandogan

**Affiliations:** 1 The David J. Apple Center for Vision Research, Department of Ophthalmology, University of Heidelberg, 69120 Heidelberg, Germany; 2 Department of Ophthalmology, Kangbuk Samsung Hospital, Sungkyunkwan University School of Medicine, Seoul 03181, Republic of Korea; 3 Augenklinik Pallas, 4600 Olten, Switzerland

**Keywords:** intraocular lens, hydrophilic, opacification, calcification, optical quality, modulation transfer function, light transmission

## Abstract

We evaluated the influence of intraocular lens (IOL) opacification on the optical performance of explanted hydrophilic acrylic IOLs. We performed a laboratory analysis of 32 Lentis LS-502-1 (Oculentis GmbH, Berlin, Germany) IOLs, explanted due to opacification, in comparison with six clear unused samples of the same IOL model. Using an optical bench setup, we obtained modulation transfer function (MTF), Strehl ratio, two-dimensional MTF, and United States Air Force (USAF) chart images. In addition, we assessed light transmission through the IOLs. The MTF values of opacified IOLs at 3-mm aperture were similar to those of clear lenses, with the median (interquartile range) values of 0.74 (0.01) vs. 0.76 (0.03) at the spatial frequency of 50 line pairs per millimeter in clear and opacified IOLs, respectively. The Strehl ratio of opacified lenses was not lower than that of clear lenses. The USAF-chart analysis showed a considerable reduction in brightness in opacified IOLs. The median (interquartile range) relative light transmission of opacified IOLs in comparison to clear lenses was 55.6% (20.8%) at the aperture size of 3 mm. In conclusion, the explanted opacified IOLs had comparable MTF values to those of clear lenses but significantly reduced light transmission.

## 1. Introduction

Most current intraocular lens (IOL) models are produced from hydrophobic or hydrophilic acrylate [1]. Higher water content of hydrophilic material allows higher IOL flexibility in comparison to hydrophobic lenses, minimizing the incision size required for the IOL implantation [1]. However, one of the main risks of hydrophilic acrylic lenses is the possibility of calcium phosphate deposit formation, which causes IOL opacification [2].

In such cases, both primary and secondary IOL calcification have been identified [3]. The latter occurs following intraocular procedures, especially involving an injection of gas or air, or is caused by an ocular or systemic illness [4,5]. While some authors recommend avoiding the implantation of hydrophilic IOLs in patients undergoing corneal or vitreoretinal surgeries involving intraocular gas or air, hydrophilic IOLs are generally deemed safe for most patients since the rate of IOL calcification is low [5]. The primary calcification, on the other hand, is related to the IOL material itself [3,4]. Due to differences in materials and manufacturing processes, certain materials are more likely to opacify than others [4]. One of the best-known examples of primary calcification is the one of Lentis HydroSmart (Oculentis GmbH, Berlin, Germany) IOL models [6]. Analysis performed by the manufacturer suggested that a possible cause for the IOL calcification is the phosphate remnants from a detergent used in the cleaning process of the IOLs, which made the manufactured IOLs more prone to the calcification [7,8]. The issue reportedly affected the IOLs manufactured between May/June 2009 and May 2015, but it remains relevant as the patients implanted with these lenses are still presenting in ophthalmology clinics for treatment [6,8,9]. In cases when patient complaints are caused by an opacified lens, the IOL explantation with implantation of a new clear IOL is the only treatment option to improve the vision.

In order to expand our understanding of the impact of IOL opacification on the patient’s vision, explanted opacified IOLs can be studied in a laboratory setting, which allows objective optical quality evaluation. Previous studies on several different opacified IOL models reported highly variable results, indicating the need for further research [2,10,11,12]. Understanding the effects of the primary calcification on the optical properties of the Lentis LS-502-1 (Oculentis) IOL is of particular importance, as this lens is among the most-commonly explanted IOLs due to opacification [6,7,13,14]. In our study, we compared the explanted opacified Lentis LS-502-1 IOLs with the clear samples of the same model in terms of optical quality and light transmission.

## 2. Materials and Methods

### 2.1. Intraocular Lenses

In this laboratory study, we analyzed 32 opacified Lentis LS-502-1 hydrophilic acrylic single-piece IOLs with a hydrophobic surface coating. The lens has a water content of 25% and a refractive index of 1.46 and features a biconvex spherical design of the optic and C-loop haptics [15]. The IOLs had been explanted because of vision complaints and sent to our laboratory for analysis. There were 4 opacified lenses with the nominal refractive power of 19.5 diopters (D), 4 lenses of 20.0 D, 8 lenses of 21.5 D, 5 lenses of 22.5 D, 6 lenses of 23.0 D, and 5 lenses of 24.5 D. We compared the optical bench measurements of these opacified IOLs with the ones of six clear, implantation-ready counterparts with equivalent nominal refractive powers.

### 2.2. Light Microscopy

Before the optical quality measurement, the opacified IOLs were inspected under the BX50 (Olympus Corporation, Tokyo, Japan) light microscope and photographed using the C-7070 (Olympus Corporation, Tokyo, Japan) camera.

### 2.3. Optical Quality Assessment

The optical quality was assessed using the OptiSpheric IOL PRO (Trioptics GmbH, Wedel, Germany) optical bench with a narrow band interferential filter of 546 nm. This optical bench setup was already described in detail in a previous publication [16]. Each IOL was placed into an IOL holder and inserted into the model eye, filled with saline solution. The model cornea used in the study had no spherical aberration.

We measured modulation transfer function (MTF), which is widely used to evaluate the optical performance of IOLs [2,17,18]. The MTF was obtained at the spatial frequencies of 25, 50 and 100 line pairs per millimeter (lp/mm) at the best focus of the IOL, at the aperture sizes of 3.0 mm and 3.75 mm. The sagittal and tangential values of the MTF were averaged. We also analyzed the Strehl ratio, which is the ratio of the area under the MTF curve compared to a perfect diffraction-limited lens and is independent of IOL refractive power [17,18]. In addition, the two-dimensional modulation transfer function (2D-MTF) was obtained at the aperture size of 3.0 mm using the same exposure settings for clear and opacified IOLs, as well as using adjusted exposure for opacified IOLs in order to achieve the same values at the center of the 2D-MTF as in clear IOLs. Furthermore, the United States Air Force (USAF) resolution test chart images were obtained through clear and opacified lenses using the same exposure settings. The brightness of the images taken through opacified IOLs was then adjusted to match the images taken through clear IOLs. Finally, we calculated the IOLs’ relative light transmission as a percentage of the light transmitted through an opacified IOL in comparison with that through a clear IOL of identical nominal refractive power.

### 2.4. Data Analysis

We performed the data analysis using Microsoft Excel 365 (Microsoft Corporation, Redmond, WA, USA) and IBM SPSS Statistics Version 28 (International Business Machines Corporation, Armonk, NY, USA). We compared the values of the opacified IOLs with those of the clear IOLs using Mann-Whitney-U test. As four comparisons were performed, the *p* value of <0.0125 was considered statistically significant according to the Bonferroni’s correction. The data are presented as medians with interquartile ranges (IQR).

## 3. Results

**Figure 1:**
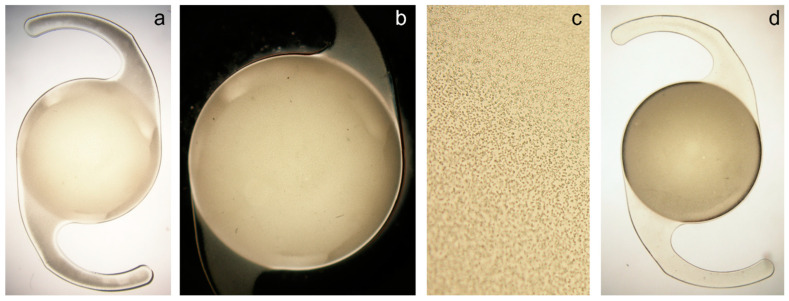
Light microscopy photographs of explanted opacified Lentis LS-502-1 IOLs. An example of a 24.5 D IOL (**a**–**c**) with a uniform calcification, resulting in reduced light transmission (relative transmission of 66.1%) but only slightly lower MTF values (Strehl ratio of 0.76 vs. 0.86 of a clear 24.5 D IOL at 3-mm aperture): (**a**)—opacified IOL optic and haptics seen against a bright background, (**b**)—photography in retroillumination against a dark background, (**c**)—under 20-fold magnification, evenly-distributed deposits can be visualized. An example of a 20.0 D IOL (**d**) with a dense opacification, which strongly reduced light transmission (relative transmission 41.9%), but only slightly affected the MTF values (Strehl ratio of 0.79 vs. 0.89 of a clear 20.0 D lens at 3-mm aperture).

The microscopic examination of the IOLs revealed diffuse, mostly evenly distributed granular deposits in the IOL optic and the haptics (Figure 1). Although some samples had their haptics amputated during the explantation, no relevant damage to the IOL optic was observed.

**Figure 2:**
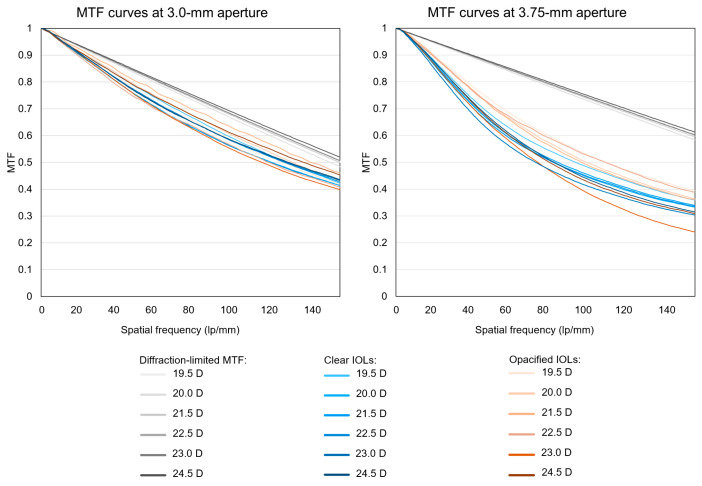
Modulation transfer function (MTF) curves of clear vs. opacified IOLs with different refractive powers.

**Figure 3:**
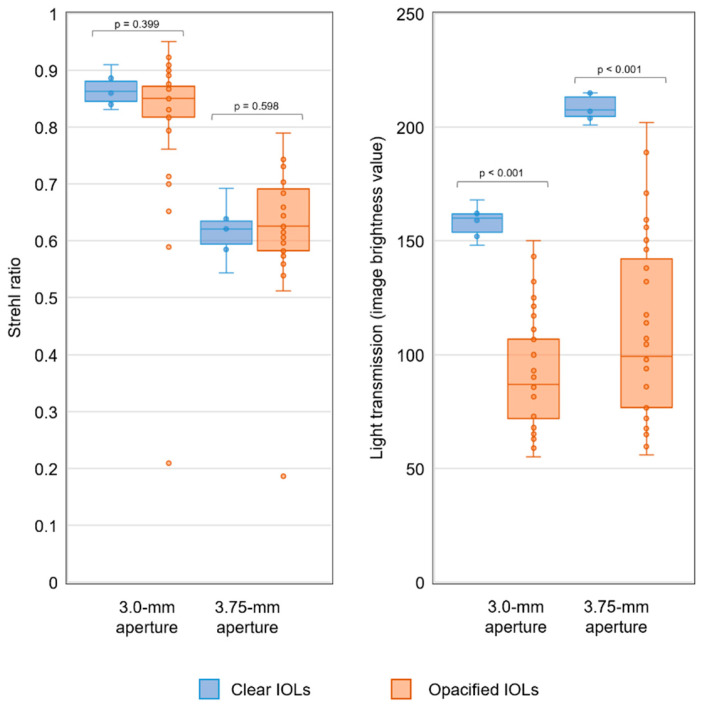
Box plots of the Strehl ratio values (graph on the **left**) and light transmission (graph on the **right**) in clear and opacified IOLs.

The MTF values of most of the opacified IOLs were comparable to those of clear lenses, as indicated by almost identical median (IQR) values between the two groups at the aperture size of 3.0 mm: 0.87 (0.01) vs. 0.88 (0.02), 0.74 (0.01) vs. 0.76 (0.03), and 0.56 (0.02) vs. 0.57 (0.05) at the spatial frequencies of 25 lp/mm, 50 lp/mm, and 100 lp/mm, in the clear and opacified IOLs, respectively. At 3.75-mm aperture, they were 0.81 (0.02) vs. 0.84 (0.04) at the spatial frequency of 25 lp/mm, 0.62 (0.03) vs. 0.68 (0.06) at 50 lp/mm, and 0.42 (0.02) vs. 0.47 (0.07) at 100 lp/mm, in the clear and opacified IOLs, respectively. MTF curves of representative IOL samples are shown in Figure 2. The median (IQR) Strehl ratio of the opacified lenses was within the range of the clear lenses both at the 3-mm aperture (0.86 (0.04) of the clear IOLs vs. 0.85 (0.05) of the opacified IOLs, *p* = 0.399) and at the 3.75-mm aperture (0.62 (0.04) of the clear IOLs vs. 0.63 (0.11) of the opacified IOLs, *p* = 0.598). However, inter-sample variation was higher in the group of the opacified IOLs, with some IOLs having considerably lower values (Figure 3). At the 3-mm aperture, 10 samples of the opacified IOLs (31.3%) had Strehl ratio values of less than 0.83, which was the lowest value observed in a clear IOL. The values of the MTF and Strehl ratio in samples grouped by their nominal refractive powers are shown in Table 1.

**Figure 4:**
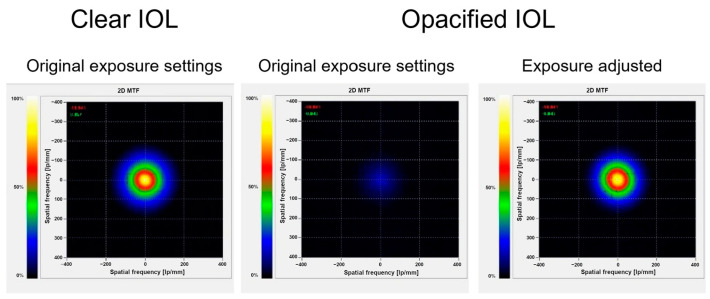
Two-dimensional modulation transfer function (2D-MTF) obtained at a 3-mm aperture through a clear (**left**) and an opacified (**middle**) 23.0 D IOL using the same exposure settings, as well as through the opacified IOL after the exposure adjustment (**right**).

**Figure 5:**
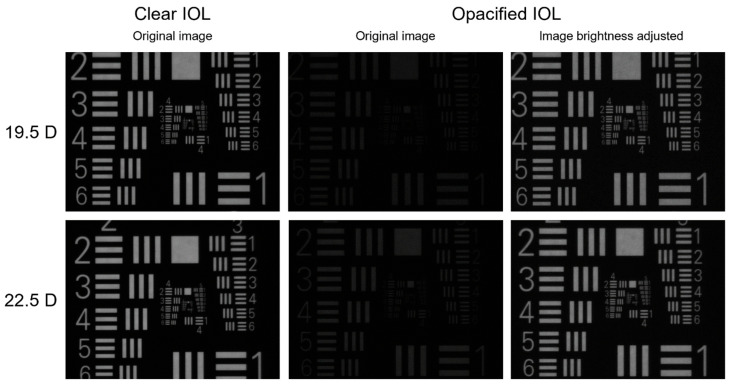
Examples of USAF target images obtained through clear IOLs (**left column**) and opacified IOLs (**middle column**) at the same exposure conditions reveal a significant light loss due to opacification. After adjusting the image brightness to match that of clear IOL, however, the image-quality difference between the clear and opacified IOLs becomes unnoticeable (**right column**).

The opacified IOLs transmitted considerably less light in comparison to the clear IOLs. The median (IQR) image brightness value was 160 (8) vs. 87 (35) at the aperture of 3 mm (*p* < 0.001) and 208 (9) vs. 99 (68) at 3.75 mm (*p* < 0.001) in clear and opacified lenses, respectively. The median (IQR) relative light transmission of opacified IOLs was 55.6% (20.8%) at the aperture size of 3.0 mm and 47.1% (29.3%) at 3.75 mm, in comparison to that of clear IOLs of equivalent nominal refractive power. The relative light transmission was less than 80% in 28 (87.5%) opacified IOLs and less than 60% in 19 (59.4%) lenses at the 3-mm aperture. Using equivalent exposure settings, the 2D-MTF was significantly reduced in opacified lenses. After adjusting the exposure, however, the 2D-MTF of opacified and clear IOLs were comparable (Figure 4). While the USAF-target analysis showed a considerable reduction in brightness in opacified IOLs, the image resolution was similar to the one of clear IOLs. After adjusting the brightness, the images obtained through clear and opacified IOLs were almost identical (Figure 5).

## 4. Discussion

Primary IOL calcification is generally considered to be a rare complication, but it occurs more commonly in certain IOL models [14,19]. The Hydroview H60M IOL (Bausch & Lomb, Rochester, NY, USA) is a well-documented example of a lens prone to primary calcification, with a reported opacification rate of up to 15.4% after a follow-up of 3 years [20,21]. More recently, the cases of the opacified LS-502-1, the IOL examined in this study, were observed, with a prevalence of up to 53.3% at 5 years postoperatively [9,22]. Due to the popularity of this IOL model and its high rate of opacification, it is among the most-commonly explanted IOLs due to opacification [6,7,13,14]. In a study by Neuhann et al. that analyzed 200 explanted lenses, the IOL opacification was found to be the cause for explantation in three-quarters (76.5%) of the cases, and the LS-502-1 IOL was the most commonly explanted lens model (21.5% of all explanted IOLs) [6].

In most cases, the only option to improve the vision in a patient with an opaque lens is the IOL-exchange surgery. Gurabardhi et al. presented a series of 63 patients who underwent the explantation of the opacified Lentis IOLs on average 49 ± 14 months after the cataract surgery [7]. The authors reported significantly improved corrected distance visual acuity after the surgery [7]. Although the rate of vision-threatening complications was low, the IOL had to be explanted together with the capsular bag in 52% of the cases due to strong fibrotic adhesions, and anterior vitrectomy had to be performed in 65% of the surgeries [7]. Goemaere et al. analyzed the IOL-exchange indications in 492 eyes over 15 years. The IOL opacification was found to be the most common reason for the IOL-exchange surgery, with the opacified IOLs explanted after the mean of 60 ± 38 months following their implantation [13]. A study by Gashau et al. reported the exchange of opacified IOLs to improve the quality of life and visual acuity in most patients but warned that the patients need to be extensively informed about the complication risk as the outcomes in some patients were poor [23]. Dagres et al. reported that only 52% of the IOL-exchange surgeries were uneventful, with complications such as posterior capsule rupture and zonular dehiscence occurring in the remaining cases [24]. It is also important not to perform the Nd:YAG laser capsulotomy in case of IOL opacification as it can further increase the intraoperative complication risk [2,25]. Leysen et al. found the presence of the Nd:YAG laser capsulotomy to correlate strongly with vitreous loss requiring anterior vitrectomy [26]. The indication for IOL-exchange surgery always needs to be clearly evaluated and the decision can be especially difficult in cases when visual acuity is still good.

Laboratory analyses of explanted opacified hydrophilic IOLs revealed the presence of fine granular calcium phosphate deposits, which can be stained with alizarin red and von Kossa stains [2,6,7,14,27]. Gartaganis et al. investigated the biochemical mechanisms that are responsible for the calcification of the hydrophilic acrylic IOLs by analyzing 30 explanted opacified IOLs [28]. They identified the plate-like octacalcium phosphate crystallites and the prismatic hydroxyapatite crystallites, the latter being the predominant crystalline phase in the opacified lenses [28]. The authors also performed aqueous humor analysis and found it to be supersaturated with respect to calcium phosphates, while the surface hydroxyl groups of the IOL materials facilitated surface nucleation and crystal growth [28,29]. Drimtzias et al. showed that the calcification starts in the interior of the lens, due to calcium and phosphate ion diffusion into the lens [29]. In addition to the proposed mechanism, the possibility of other mechanisms of IOL calcification cannot be excluded, as the study by Lai et al. reported the varying morphology and chemical constitution of the deposits between two samples of the same IOL model (Hydroview H60M), where fluorine, magnesium, and sodium were found in addition to calcium and phosphorus in one sample [30]. Although calcification can occur in various hydrophilic acrylic lenses, it occurred more frequently in the Lentis IOLs: the issue that the manufacturer first attributed to the system of lens packaging [31]. However, the later analysis indicated that the phosphate remnants from a detergent used to clean the IOLs were the possible cause for the high calcification rate of the Lentis IOLs [7,8]. Using scanning electron microscopy, it was shown that these crystalline-like deposits are distributed in a line parallel to the anterior and posterior IOL surface [2]. They are found up to 100 µm under the IOL surface [32]. Gartaganis et al. performed a detailed scanning electron microscopy analysis of 6 opacified samples of the same lens model as the one in our study (Lentis LS-502-1) and found the subsurface formation of calcium phosphate crystalline deposits, which seemed to diffuse to the IOL surface, and their clusters resulted in the formation of lumps on the surface [33]. Using this technology, other authors also reported the presence of granular deposits in the opacified Lentis IOLs [31,34,35,36,37]. In contrast to the secondary IOL calcification after intraocular procedures, where the IOL opacification is usually limited to the central area of the lens optic, the primary IOL opacification is usually homogenous [10,14,27,32]. In this study, we also observed a homogenous pattern, with granular deposits in the optic and the haptics of the studied lenses, although the haptics appeared to be less affected in some cases.

Laboratory analysis of the optical properties of opacified IOLs can improve our understanding of how the lens performance is affected. This type of analysis is generally difficult because the IOLs sometimes need to be cut into halves during the explantation process, rendering optical bench analysis of the explants impossible. In this study, however, all the IOLs had been explanted leaving the optic intact, which enabled optical quality measurements.

Although the optical characteristics of opacified hydrophilic IOLs had been analyzed by other studies before, discrepancies exist regarding the effect that IOL calcification has on their optical quality [2,10,11,12]. Łabuz et al. performed an optical bench analysis of opacified IOLs and observed a significant reduction of the MTF values in the IOLs with a localized (secondary) calcification, whereas the MTF was almost unaffected by a homogenous (primary) opacification [10]. In contrast, the study by Werner et al. found decreased MTF values in all the samples (*n* = 13) of different IOL models explanted because of calcification [12]. The authors did not explicitly state if the calcification in the examined IOLs was primary or secondary, but the representative images of the IOLs showed a homogenous opacification pattern, which did not significantly affect the MTF in the study by Łabuz et al. [10,11,12]. Tandogan et al. also reported low MTF values at all spatial frequencies in an explanted opacified Euromaxx ALI313Y (Argonoptics, Haltern am See, Germany) IOL sample with a homogenous opacification [2]. In general, the interpretation of the results of these studies is complicated by small sample sizes and the analysis of different IOL models together. For example, the model of several of the IOLs examined in the study by Łabuz et al. was not known, with spherical, aspheric, and aberration-neutral IOLs analyzed together [10]. The variability in IOL calcification in different IOL models could have been the reason for the disagreement of the study findings, which highlights the need to analyze different IOL models separately [38]. In our study, after examining 32 samples of the same model IOL with primary homogenous calcification, we found only one sample to have very low MTF values with the Strehl ratio of 0.21 at a 3-mm aperture. We concluded that the MTF values in most of the explanted opacified Lentis LS-502-1 IOLs were comparable to those of clear lenses, which agrees with the findings by Łabuz et al. [10]. Another possible reason for discrepancies among studies is that apart from the opacification pattern, the MTF was found to be affected by the extent of the calcification [11]. Therefore, even in the case of a homogenous opacification, the MTF values could be low if the opacification is very dense [2].

Despite the largely unaffected MFT values of opacified lenses in our study, most of the opacified IOLs had significantly reduced light transmission. This effect was studied in other models of opacified hydrophilic acrylic IOLs before [12,27,39]. Michelson et al. used a spectrophotometer to measure the light transmittance in the visible light spectrum and found it to range from 79.9% to 97.3% [39]. Similar values were reported by later studies using the same methodology [12,27]. The average light transmittance ranged between 82.6% and 90.3% [12,27,39]. We used a different methodology as we did not directly measure the light transmission in opacified IOLs but calculated relative transmission in comparison to clear IOLs, based on the brightness values of the pixels, as detected by the camera of the optical bench. This setting demonstrated a nearly proportional relationship with in vitro glare assessment in opacified IOL after intraocular gas injection [11]. Our relative transmission values were lower in comparison to the light transmittance reported in the studies mentioned above, which could be due to different measurement technology (optical bench setup vs. spectrophotometer), different wavelengths used (monochromatic vs. polychromatic light), or different extent of IOL opacification. In addition to the relative transmission measurements, we could visualize the reduction in brightness by obtaining the USAF target images. Although the image quality seemed to be unaffected after the brightness was adjusted, visual acuity could still be affected because visual acuity varies with illumination [10,40]. In very bright light, visual acuity does not depend on illumination, but at lower intensities, a rapid reduction of visual acuity occurs [40]. Therefore, while a patient with an opacified IOL might not notice the light loss in good lighting conditions, e.g., during visual-acuity testing, the loss of brightness should result in a reduction of visual acuity in dimmer light.

The light loss in IOL calcification is primarily the result of light scattering by the calcium phosphate particles [10,11,12,15]. Studies using Scheimpflug photography found very high levels of backward light scattering in opacified lenses [12,27,39]. This light is reflected back, does not contribute to the retinal image creation, and is therefore lost. In addition, IOL calcification causes forward light scattering [10,12,15]. This light reaches the retina and is responsible for disability glare [41]. Son et al. used a ray propagation imaging technique and could visualize forward light scattering in opacified IOLs as the background haze around the central beam [42]. The amount of the forward-scattered light can be described using the straylight metric and measured with the C-Quant (Oculus, Wetzlar, Germany) device both in clinical and laboratory settings [10,42]. The straylight metric is independent of visual acuity [41]. While aberrations influence the peak of the point spread function (PSF) and cause a reduction of visual acuity, the straylight corresponds to the peripheral part of the PSF and causes complaints such as glare and hazy vision [10,41]. Straylight also increases the halo size and the luminance detection threshold and decreases contrast sensitivity [43]. A normal pseudophakic eye was reported to have a mean straylight value of 1.21 ± 0.21 log units [44]. Values above 1.47 log units cause serious visual restriction [45]. Opacified hydrophilic acrylic IOLs were found to have straylight values of 1.79 ± 0.37 log units on average, in comparison to only 0.36 ± 0.05 log units in clear IOLs [12]. Łabuz et al. compared the straylight in IOLs with a homogenous vs. localized opacification and found significantly higher values in the lenses with a homogenous opacification pattern [10]. The median straylight value was 2.26 log units in IOLs with a homogenous opacification and 1.84 log units in those with a localized opacification [10]. The authors associated the amount of straylight with the number and size of the calcium granules in the opacified IOLs [10,11]. In the current study, the straylight metric was not assessed, which is a limitation of our work. We would expect, however, elevated straylight values in the studied lenses due to their homogenous opacification pattern and high light-transmission loss.

Clinical observations correspond to the conclusions of laboratory studies of the explanted opacified lenses. A study by Blundell et al. compared objective clinical metrics and subjective complaints of patients with opacified vs. clear IOLs of the same model (Hydroview) and found the patients with opacified lenses to have dramatically higher levels of glare, whereas visual acuity was only mildly affected [46]. Furthermore, the vision-related quality of life was also diminished in the opacified-IOL group [46]. For instance, while most of the patients with clear IOLs did not complain of misty vision (median questionnaire score of 0, range 0–5), those with opacified IOLs had a median score of 3 (range 0–5) [46]. It is therefore important to consider that subjective patient complaints may, in some cases, be a sufficient reason to explant an opacified IOL even when visual acuity is still good.

In conclusion, the explanted opacified IOLs had comparable MTF values to those of the clear lenses but significantly reduced light transmission. Our findings suggest that the patients with primary IOL calcification could have good visual acuity in bright lighting conditions but experience difficulties in lower-light levels. Further studies are needed to analyze the relationship between the optical bench analysis results and clinical metrics in patients with opacified IOLs, such as how the low-light visual acuity is affected by the reduced light transmission in patients with IOL opacification.

## Figures and Tables

**Table 1:** Modulation transfer function, Strehl ratio, and relative light transmission in clear and opacified intraocular lenses.

IOL Refractive Power (D)	IOL Clarity	Number of Samples	3-mm Aperture					3.75-mm Aperture				
			MTF @ 25 lp/mm, Median Value (IQR)	MTF @ 50 lp/mm, Median Value (IQR)	MTF @ 100 lp/mm, Median Value (IQR)	Strehl Ratio, Median Value (IQR)	Relative Light Transmission (Percentage), Median Value (IQR)	MTF @ 25 lp/mm, Median Value (IQR)	MTF @ 50 lp/mm, Median Value (IQR)	MTF @ 100 lp/mm, Median Value (IQR)	Strehl Ratio, Median Value (IQR)	Relative Light Transmission (Percentage), Median Value (IQR)
19.5	clear	1	0.89	0.77	0.59	0.91	100	0.86	0.70	0.48	0.69	100
	opacified	4	0.88 (0.07)	0.76 (0.08)	0.55 (0.07)	0.82 (0.05)	48.0 (10.9)	0.85 (0.02)	0.68 (0.01)	0.47 (0.01)	0.59 (0.05)	39.30 (12.44)
20.0	clear	1	0.87	0.74	0.55	0.89	100	0.81	0.62	0.41	0.64	100
	opacified	4	0.90 (0.03)	0.79 (0.04)	0.60 (0.03)	0.83 (0.10)	46.7 (5.0)	0.88 (0.05)	0.75 (0.09)	0.54 (0.10)	0.69 (0.04)	37.70 (2.41)
21.5	clear	1	0.87	0.74	0.54	0.83	100	0.82	0.63	0.44	0.62	100
	opacified	8	0.88 (0.02)	0.76 (0.02)	0.57 (0.03)	0.87 (0.13)	55.7 (17.3)	0.84 (0.03)	0.68 (0.06)	0.47 (0.05)	0.65 (0.08)	46.13 (24.50)
22.5	clear	1	0.87	0.74	0.55	0.84	100	0.80	0.60	0.42	0.59	100
	opacified	5	0.88 (0.01)	0.76 (0.03)	0.59 (0.06)	0.87 (0.04)	62.1 (16.1)	0.84 (0.02)	0.68 (0.04)	0.45 (0.06)	0.66 (0.08)	56.68 (22.12)
23.0	clear	1	0.87	0.75	0.56	0.87	100	0.77	0.56	0.39	0.54	100
	opacified	6	0.88 (0.02)	0.76 (0.02)	0.56 (0.01)	0.86 (0.03)	61.7 (23.5)	0.83 (0.03)	0.65 (0.05)	0.44 (0.07)	0.63 (0.03)	64.19 (33.35)
24.5	clear	1	0.88	0.76	0.57	0.86	100	0.83	0.64	0.43	0.62	100
	opacified	5	0.87 (0.03)	0.75 (0.06)	0.56 (0.06)	0.82 (0.08)	63.5 (14.2)	0.80 (0.01)	0.59 (0.01)	0.39 (0.01)	0.56 (0.04)	61.40 (18.19)
**all** **refractive powers**	**clear**	**6**	**0.87 (0.01)**	**0.74 (0.01)**	**0.56 (0.02)**	**0.86 (0.04)**	**100 (0)**	**0.81 (0.02)**	**0.62 (0.03)**	**0.42 (0.02)**	**0.62 (0.04)**	**100 (0)**
	**opacified**	**32**	**0.88 (0.02)**	**0.76 (0.03)**	**0.57 (0.05)**	**0.85 (0.05)**	**55.6 (20.8)**	**0.84 (0.04)**	**0.68 (0.06)**	**0.47 (0.07)**	**0.63 (0.11)**	**47.07 (29.25)**

D—diopters; IOL—intraocular lens; IQR—interquartile range; lp/mm—line pairs per millimeter; MTF—modulation transfer function.

## Data Availability

All data generated or analyzed during this study are included in this published article.

## References

[1] Auffarth G.U., Apple D.J. (2001). History of the development of intraocular lenses. Ophthalmologe.

[2] Tandogan T., Khoramnia R., Choi C.Y., Scheuerle A., Wenzel M., Hugger P., Auffarth G.U. (2015). Optical and material analysis of opacified hydrophilic intraocular lenses after explantation: A laboratory study. BMC Ophthalmol..

[3] Neuhann I.M., Kleinmann G., Apple D.J. (2008). A New Classification of Calcification of Intraocular Lenses. Ophthalmology.

[4] Britz L., Schickhardt S.K., Yildirim T.M., Auffarth G.U., Lieberwirth I., Khoramnia R. (2022). Development of a standardized in vitro model to reproduce hydrophilic acrylic intraocular lens calcification. Sci. Rep..

[5] Belin P.J., Kim J.H., Sheikh A., Winokur J., Rhee D., Deramo V. (2021). Incidence and risk of scleral-fixated Akreos (AO60) lens opacifi-cation: A case series. J. VitreoRetinal Dis..

[6] Neuhann T., Yildirim T.M., Son H.-S., Merz P.R., Khoramnia R., Auffarth G.U. (2020). Reasons for explantation, demographics, and material analysis of 200 intraocular lens explants. J. Cataract Refract. Surg..

[7] Gurabardhi M., Häberle H., Aurich H., Werner L., Pham D.-T. (2018). Serial intraocular lens opacifications of different designs from the same manufacturer: Clinical and light microscopic results of 71 explant cases. J. Cataract Refract. Surg..

[8] Oculentis B.V. Urgent Field Safety Notice for Lentis L or LS or LU by Oculentis BV. https://www.bfarm.de/SharedDocs/Kundeninfos/EN/11/2017/09163-17_kundeninfo_en.pdf;jsessionid=92D992D14BE58D1D3D761D87295A903D.intranet262?__blob=publicationFile.

[9] Scherer N.C.D., Müller K.M., Prahs P.M., Radeck V., Helbig H., Märker D.A. (2020). Serial opacification of a hydrophilic–hydrophobic acrylic intraocular lens: Analysis of potential risk factors. J. Cataract Refract. Surg..

[10] Łabuz G., Yildirim T.M., Khoramnia R., Son H.-S., Auffarth G.U. (2020). Optical function of intraocular lenses in different opacification patterns: Metrology analysis of 67 explants. J. Cataract Refract. Surg..

[11] Łabuz G., Yildirim T.M., van den Berg T.J.T.P., Khoramnia R., Auffarth G.U. (2018). Assessment of straylight and the modulation transfer function of intraocular lenses with centrally localized opacification associated with the intraocular injection of gas. J. Cataract Refract. Surg..

[12] Werner L., Stover J.C., Schwiegerling J., Das K.K. (2016). Effects of Intraocular Lens Opacification on Light Scatter, Stray Light, and Overall Optical Quality/Performance. Investig. Opthalmol. Vis. Sci..

[13] Goemaere J., Trigaux C., Denissen L., Dragnea D., Hua M.-T., Tassignon M.-J., Dhubhghaill S.N. (2020). Fifteen years of IOL exchange: Indications, outcomes, and complications. J. Cataract Refract. Surg..

[14] Khoramnia R., Yildirim T.M., Łabuz G., Mayer C.S., Auffarth G.U. (2021). Opacification of intraocular lenses: Laboratory and clinical findings. Ophthalmologe.

[15] Łabuz G., Yildirim T.M., Auffarth G.U., Son H.-S., Khoramnia R. (2021). Laboratory evaluation of higher-order aberrations and light scattering in explanted opacified intraocular lenses. Eye Vis..

[16] Tandogan T., Auffarth G.U., Choi C.Y., Liebing S., Mayer C., Khoramnia R. (2017). In vitro comparative optical bench analysis of a spherical and aspheric optic design of the same IOL model. BMC Ophthalmol..

[17] Holladay J.T., van Dijk H., Lang A., Portney V., Willis T.R., Sun R., Oksman H.C. (1990). Optical performance of multifocal intraocular lenses. J. Cataract Refract. Surg..

[18] Rawer R., Stork W., Spraul C.W., Lingenfelder C. (2005). Imaging quality of intraocular lenses. J. Cataract Refract. Surg..

[19] Khoramnia R., Salgado J., Auffarth G., Schmidt S., Wegner A., Kobuch K., Von Mohrenfels C.W. (2012). Eintrübung einer hydrophilen Intraokularlinse 4 Jahre nach Kataraktoperation. Der. Ophthalmol..

[20] Balasubramaniam C., Goodfellow J., Price N., Kirkpatrick N. (2006). Opacification of the Hydroview H60M intraocular lens: Total patient recall. J. Cataract Refract. Surg..

[21] Sher J.H., Gooi P., Dubinski W., Brownstein S., El-Defrawy S., Nash W.A. (2008). Comparison of the incidence of opacification of Hy-droview hydrogel intraocular lenses with the ophthalmic viscosurgical device used during surgery. J. Cataract Refract. Surg..

[22] Costa J.F., Bompastor-Ramos P., Marques M., Henriques J., Póvoa J., Lobo C., Alió J.L., Werner L., Murta J. (2020). Large-scale opacification of a hydro-philic/hydrophobic intraocular lens. Eur. J. Ophthalmol..

[23] Gashau A.G., Anand A., Chawdhary S. (2006). Hydrophilic acrylic intraocular lens exchange: Five-year experience. J. Cataract Refract. Surg..

[24] Dagres E., Khan M.A., Kyle G.M., Clark D. (2004). Perioperative complications of intraocular lens exchange in patients with opacified Aqua-Sense lenses. J. Cataract Refract. Surg..

[25] Grzybowski A., Markeviciute A., Zemaitiene R. (2020). A narrative review of intraocular lens opacifications: Update Ann. Transl. Med..

[26] Leysen I., Bartholomeeusen E., Coeckelbergh T., Tassignon M.J. (2009). Surgical outcomes of intraocular lens exchange: Five-year study. J. Cataract Refract. Surg..

[27] Barra D., Werner L., Costa J.L.P., Morris C., Ribeiro T., Ventura B.V., Dornelles F. (2014). Light scattering and light transmittance in a series of calcified single-piece hydrophilic acrylic intraocular lenses of the same design. J. Cataract Refract. Surg..

[28] Gartaganis S.P., Kanellopoulou D.G., Mela E.K., Panteli V.S., Koutsoukos P.G. (2008). Opacification of Hydrophilic Acrylic Intraocular Lens Attributable to Calcification: Investigation on Mechanism. Am. J. Ophthalmol..

[29] Drimtzias E.G., Rokidi S.G., Gartaganis S.P., Koutsoukos P.G. (2011). Experimental Investigation on Mechanism of Hydrophilic Acrylic Intraocular Lens Calcification. Am. J. Ophthalmol..

[30] Lai J.-Y., Chen K.-H., Hsu W.-M., Lee T.-H., Lin S.-Y. (2005). Multiple Elements in the Deposits of Opacified Hydroview Intraocular Lens. Am. J. Ophthalmol..

[31] Cavallini G.M., Volante V., Campi L., De Maria M., Fornasari E., Urso G. (2017). Postoperative diffuse opacification of a hydrophilic acrylic intraocular lens: Analysis of an explant. Int. Ophthalmol..

[32] Yildirim T.M., Auffarth G.U., Łabuz G., Bopp S., Son H.-S., Khoramnia R. (2018). Material Analysis and Optical Quality Assessment of Opacified Hydrophilic Acrylic Intraocular Lenses After Pars Plana Vitrectomy. Am. J. Ophthalmol..

[33] Gartaganis S.P., Prahs P., Lazari E.D., Gartaganis P.S., Helbig H., Koutsoukos P.G. (2016). Calcification of Hydrophilic Acrylic Intraocular Lenses with a Hydrophobic Surface: Laboratory Analysis of 6 Cases. Am. J. Ophthalmol..

[34] Bang S.P., Moon K., Lee J.H., Jun J.H., Joo C.K. (2019). Subsurface calcification of hydrophilic refractive multifocal intraocular lenses with a hydrophobic surface: A case series. Medicine.

[35] Bompastor-Ramos P., Póvoa J., Lobo C., Rodriguez A.E., Alió J.L., Werner L., Murta J.N. (2016). Late postoperative opacification of a hydrophilic-hydrophobic acrylic intraocular lens. J. Cataract Refract. Surg..

[36] Yamashita K., Hayashi K., Hata S. (2020). Toric Lentis Mplus intraocular lens opacification: A case report. Am. J. Ophthalmol. Case Rep..

[37] Yildirim T.M., Łabuz G., Khoramnia R., Son H.S., Schickhardt S.K., Lieberwirth I., Knorz M.C., Auffarth G.U. (2020). Impact of Primary Calcification in Segmented Refractive Bifocal Intraocular Lenses on Optical Performance Including Straylight. J. Refract. Surg..

[38] Izak A.M., Werner L., Pandey S.K., Apple D.J. (2003). Calcification of modern foldable hydrogel intraocular lens designs. Eye.

[39] Michelson J., Werner L., Ollerton A., Leishman L., Bodnar Z. (2012). Light scattering and light transmittance in intraocular lenses explanted because of optic opacification. J. Cataract Refract. Surg..

[40] Hecht S. (1928). The Relation between Visual Acuity and Illumination. J. Gen. Physiol..

[41] Van den Berg T.J.T.P. (2017). The (lack of) relation between straylight and visual acuity. Two domains of the point-spread-function. Ophthalmic Physiol. Opt..

[42] Son H.-S., Łabuz G., Khoramnia R., Yildirim T.M., Choi C.Y., Knorz M.C., Auffarth G.U. (2021). Visualization of Forward Light Scatter in Opacified Intraocular Lenses and Straylight Assessment. Diagnostics.

[43] Van der Mooren M., Rosén R., Franssen L., Lundström L., Piers P. (2016). Degradation of Visual Performance with Increasing Levels of Retinal Stray Light. Investig. Ophthalmol. Vis. Sci..

[44] Abuz G., Reus N.J., van den Berg T.J. (2015). Ocular straylight in the normal pseudophakic eye. J. Cataract Refract. Surg..

[45] Van Bree M.C., Zijlmans B.L., Van den Berg T.J. (2008). Effect of neodymium:YAG laser capsulotomy on retinal straylight values in patients with posterior capsule opacification. J. Cataract Refract. Surg..

[46] Blundell M.S., Mayer E.J., Knox Cartwright N.E., Hunt L.P., Tole D.M., Dick A.D. (2010). The effect on visual function of Hydroview intraocular lens opacification: A cross-sectional study. Eye.

